# Specific energy reduction in a semi-autogenous grinding mill circuit by an automatic control system

**DOI:** 10.1038/s41598-025-09263-w

**Published:** 2025-07-01

**Authors:** Thomás Pinto, Moisés T. da Silva, Guilherme V. Raffo, Thiago A. M. Euzébio

**Affiliations:** 1 Instituto Tecnológico Vale, Robotics, Instrumentation and Control Lab., Ouro Preto, Brazil; 2 Graduate Program in Electrical Engineering, Universidade Federal de Minas Gerais, Belo Horizonte, Brazil; 3 Department of Electronic Engineering, Universidade Federal de Minas Gerais, Belo Horizonte, Brazil; 4 Universidade Federal Rural de Pernambuco, Belo Jardim, Brazil; 5 Virtus-CC, Campina Grande, Brazil; 6 Helmholtz-Zentrum Dresden-Rossendorf, Dresden, Germany

**Keywords:** Electrical and electronic engineering, Mechanical engineering

## Abstract

Grinding operations, especially those involving semi-autogenous mills, account for a significant portion of energy use in mineral processing. In this work, we describe the application of an advanced regulatory control strategy in a copper plant aimed at improving energy efficiency through automation. The system combines cascade and feedforward control structures to attenuate variations in the mill load, a key factor influencing energy consumption and process stability. The control scheme was integrated into the plant’s existing automation infrastructure and evaluated through a three-month industrial trial. By shifting from manual to automatic regulation of the feed rate, the plant reduced the influence of process disturbances and maintained more consistent operation. The automated system achieved a 5.84% reduction in specific energy consumption and a 1.90% increase in productivity. These results demonstrate the potential of enhanced regulatory control to deliver measurable performance gains with minimal changes to existing operations.

## Introduction

Mining is a highly intense energy consuming industry. Among its common processing units, comminution processes - such as crushing and grinding - are often the most energy consumers, accounting for around 50% of a mine’s total energy consumption^1^. The role of these processes is to reduce the size of ore particles to make them manageable and facilitate the extraction of valuable minerals. However, as stated in^2^, only 9% of the energy consumed is applied to breaking the ore - the rest is lost by heat.

A compelling approach to address the existing room for improvement in energy efficiency is increasing the grinding process effectiveness using automatic control systems. For instance, in^3^, the authors have compared three different control systems designed to reduce the specific energy of a simulated grinding circuit. The authors have shown that an energy efficiency gain was observed regardless of the controller applied, ranging from 1.5 to 6.7%. The authors in^4^ have proposed an economic model predictive control (EMPC) for a simulated grinding-flotation circuit. The controller has been synthesized to control several process variables, aiming to optimize an objective function that penalizes the circuit’s power consumption. Results have indicated a reduction of specific energy consumption of 0.4 to 2.6%.

Furthermore, well designed automatic control systems show high potential for achieving good energy efficiency improvements at minimal capital expenditure, as most of the instrumentation and automation tools required are usually already in place in a processing plant^5^. Works discussing the gains in energy efficiency of industrial grinding circuits through automated control systems include^6^, where the author has proposed a fuzzy logic control to improve the performance of a semi-autogenous grinding (SAG) mill of one nickel plant. Reducing the process specific energy was one of the controller’s goals. Results from six months of application showed a reduction of 8% in the specific energy. Similarly, a fuzzy controller has been applied to improve the stability and production of a SAG mill circuit in a sulfide plant^7^. The main goal of the controller was to increase the stability of key variables, namely the mill load, power draw, and throughput. After a few months of application, the authors have reported a reduction of 40% in the mill energy consumption standard deviation. The authors in^8^ have developed an MPC to control multiple variables of a copper SAG mill circuit, including the mill load and power consumption. Application results have proved the controller efficiency, which reduced the average specific energy consumption by 4.5% and increased production by 10%.

To the best of our knowledge, the papers that describe the application of control systems in industrial grinding systems to improve their energy efficiency often apply advanced control strategies, such as fuzzy logic control and MPC. These controller strategies are attractive due to their capacity to handle multivariable, non-linear, complex systems, which are common characteristics of grinding systems. However, although these techniques yield good results, they are complex structures that require higher skills and knowledge to develop and maintain, which may not often be available in an industrial plant. On the other hand, regulatory controllers - most commonly proportional-integral-derivative (PID) controllers - are feasible options to improve an industrial process operating conditions even under changes in the operating point and inherent process disturbances. As stated in^4,9,10^, enhanced regulatory controllers such as cascade control, feedforward, and override provide some advantages in comparison to the more advanced techniques: smaller cost of implementation and maintenance, simpler to develop and tune, and operator-friendly.

In this work, we discuss the development and application of an advanced regulatory control structure to increase the energy efficiency of an industrial SAG mill circuit. The control structure is synthesized as a combination of cascade and feedforward schemes, whose main objective is to reduce the variability of key process variables. This work was conducted on a copper plant of Vale S.A., a mining company in Brazil.

## Related work

Improving the performance and energy efficiency of grinding circuits has been the focus of extensive research, particularly through the application of advanced control strategies. Model Predictive Control (MPC), fuzzy logic, and intelligent systems have demonstrated promising results in simulations. For instance, a multiobjective tuning method for MPC applied to grinding processes was proposed by Yamashita et al.^11^, showing effective particle size control and throughput improvements. However, this approach was validated only in simulation environments and did not address energy consumption directly. Similar limitations appear in the fuzzy logic self-tuning PID controller presented by Khodadadi et al.^12^, which also lacked real-world validation and energy performance metrics.

Energy consumption is more explicitly addressed in the work by Bouchard et al.^3^, who used simulation to compare PI and MPC strategies in a two-stage grinding circuit. Their study showed potential reductions in specific energy consumption ranging from 1.5% to 6.7%, highlighting the relevance of control in energy efficiency. Nonetheless, the absence of industrial deployment leaves questions about the feasibility and robustness of such solutions under real process conditions.

Other studies have explored intelligent grinding systems from a broader perspective. Quintanilla et al.^13^ developed a digital twin framework integrating expert control and recurrent neural networks, trained on industrial data but validated exclusively in simulation. Similarly, the work by Miranda-Órdenes et al.^14^ on fractional adaptive controllers and by Ziolkowski et al.^15^ on extremum seeking control both focused on robustness and tuning in nonlinear grinding circuits, yet their findings remained confined to simulation environments, without direct energy assessments.

Recent efforts have attempted to bridge industrial practice and advanced analytics. A study by Saldaña et al.^16^ applied statistical and machine learning methods to operational data from a copper mine, aiming to predict overloads and improve process stability. While grounded in real plant data, their work focused on throughput and reliability rather than direct energy savings.

In summary, while the literature demonstrates a variety of advanced control techniques and modeling frameworks for grinding processes, most remain untested in operational environments or do not explicitly quantify energy performance. The present work addresses this gap by implementing a cascade and feedforward control system in an industrial SAG mill, achieving measurable improvements in specific energy consumption and process stability under real conditions.

## Process overview

**Fig. 1:**
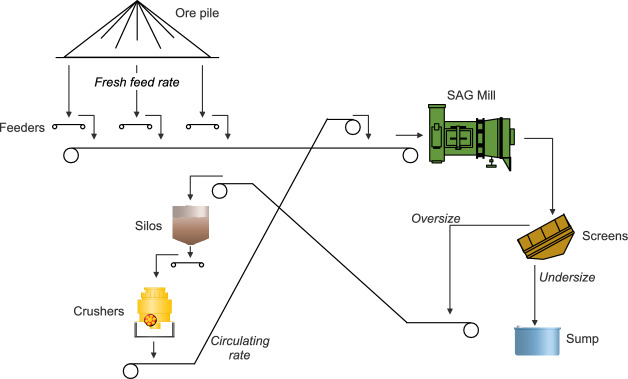
SAG mill circuit of Sossego’s plant.

The SAG mill circuit, depicted in Fig. 1, is fed by an ore pile formed by material coming from the mine. Three varying speed feeders withdraw material from the ore pile, regulating the circuit’s fresh feed rate, a portion of the total amount of material that feeds the SAG mill. Two vibrating screens classify the material discharged by the mill into two streams: *undersize* and *oversize*. The *undersize*, composed of material with a granulometry size below the screen’s mesh, is the circuit product and, therefore, is directed to the subsequent process. Conversely, the *oversize* comprises coarse material that requires another breakage stage. Thus, it is directed to crushers, whose throughput, namely the circulating rate, is redirected to the SAG mill.

The power draw of a grinding mill is heavily related to equipment factors such as torque and rotational speed, but it is also influenced by various process variables including feed size, mill load, and material characteristics^17,18^. Due to this strong correlation among variables, automatic control systems play a critical role in ensuring an energy-efficient operation. As stated in^5^, even if the energy consumption of equipment is not directly considered within the structure of an automatic control system, its energy efficiency can be enhanced by reducing the variability of key process variables associated with it. Consequently, improvements in control systems have the potential to yield significant enhancements in the energy efficiency of a grinding mill circuit. Specific energy consumption, representing the energy consumed per processed mass, serves as a common metric for assessing the energy efficiency performance of a grinding mill.

It is well established that maintaining the mill load close to an optimal value is a key enabler of both energy efficiency and throughput maximization. As demonstrated by Ziolkowski et al.^15^, grind curves—parabolic relationships between mill load and process performance—show that both throughput and power consumption reach optimal points at specific load levels, which shift based on ore characteristics. Operating away from these optimal conditions leads to inefficient breakage dynamics and increased energy demand. Similarly, the results reported in^19^ confirm that variations in mill filling have a dramatic effect on mill throughput and grind performance. Their experimental findings show that excessive filling dampens impact energy and lowers breakage rates, while underfilling results in insufficient grinding action. Therefore, the ability to stabilize the mill load dynamically around this optimum not only improves short-term process control but also establishes a robust foundation for long-term energy efficiency and operational consistency.

**Fig. 2:**
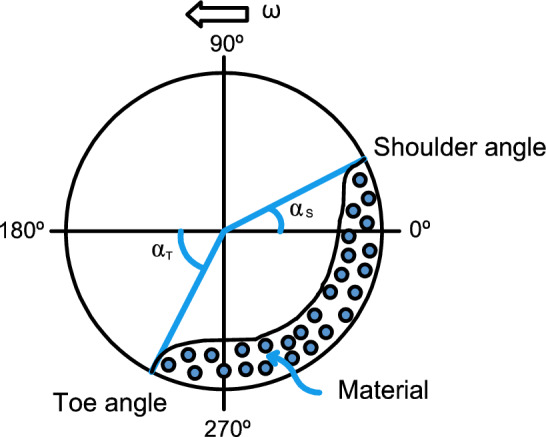
A front and internal view of a mill. The toe ($\alpha _T$) and shoulder ($\alpha _S$) angles vary as the amount of material changes.

Changes in the internal load of a SAG mill significantly affect its energy consumption. This relationship is closely linked to the dynamics of the charge inside the rotating mill. As illustrated in Fig. 2, the toe angle ($\alpha _T$) and shoulder angle ($\alpha _S$) are key geometric parameters that help describe the behavior of the mill load. The toe angle represents the lowest point at which the ore slurry settles in the mill, while the shoulder angle indicates the position where the material detaches from the shell and begins its cascading motion. Variations in the mill load alter these angles: as the load increases, more slurry accumulates at the bottom, shifting the toe further to the left (in the direction of rotation) and thereby reducing $\alpha _T$. This accumulation dampens the energy transferred to the charge, resulting in lower power draw. However, this also compromises breakage efficiency, as the reduced toe angle limits the impact energy delivered to the ore particles^20^. Thus, maintaining an optimal load is not only essential for energy efficiency but also for preserving grinding performance.

## Proposed control system

**Fig. 3:**
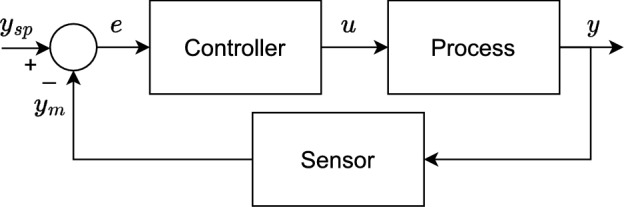
Generic representation of a feedback closed-loop system. The variables *y* and $y_{sp}$ are the controlled variable and corresponding setpoint, respectively; $y_m$ is the measured value of the controlled variable, here assumed $y_m=y$; *e* is the error between the setpoint and the controlled variable; and *u* is the control action.

A generic representation of the feedback closed-loop system is illustrated in Fig. 3. As a regulatory control system, the PID controller operates by adjusting a manipulated variable to steer a controlled variable, maintaining it close to a given setpoint. It follows the feedback principle, meaning that the error between the controlled variable and the setpoint is continuously evaluated and used to calculate its control actions as follows

1 $$\begin{aligned} u(t)=K_p\left( e(t)+\frac{1}{T_i}\int _0^te(t)dt + T_d \frac{de(t)}{dt} \right) , \end{aligned}$$

in which, *t* denotes time, $K_p$ is the proportional gain, and $T_i$ and $T_d$ are the integral and derivative times, respectively.

An advanced regulatory control (ARC) system is a combination of elements that enhance the capability of a single feedback PID control loop to deal with complex multivariable nonlinear industrial processes^21^. The ARC structure used in this work combines the cascade and feedforward elements.

### Cascade controller

**Fig. 4:**
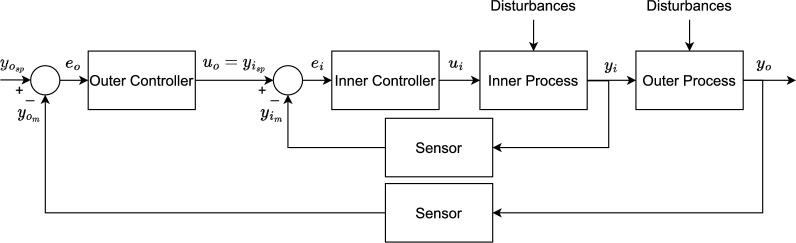
Typical structure of a cascade controller. The variables with subscripts *o* and *i* are associated with the outer and inner loops, respectively.

A typical cascade control scheme employs multiple nested feedback control loops. Figure 4 illustrates the case for a two-loop scheme. This structure helps attenuate unmeasured disturbances before they affect the process output, especially when disturbances are associated with the manipulated variables^22^. By employing an inner loop, the inner controller regulates its associated controlled variable fast to avoid disturbing the controlled variable of the outer loop. Meanwhile, the outer controller is responsible for defining the setpoint of the inner controller to ensure proper control of its associated controlled variable.

### Feedforward controller

**Fig. 5:**
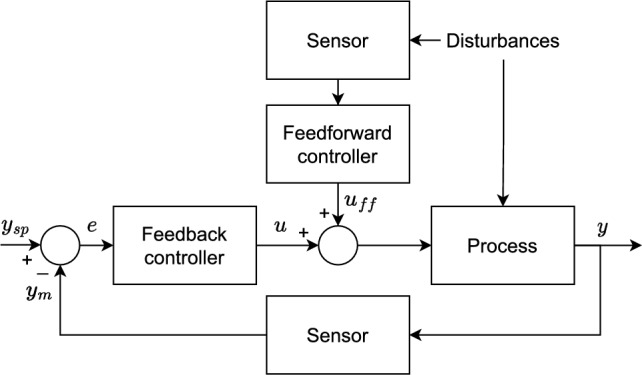
Typical structure of a feedforward controller.

The purpose of a feedforward controller is to mitigate the effect of measured disturbances, anticipating their impact on the controlled variables of a process. These controllers typically complement feedback controllers, compensating the latter’s control actions to account for the measured disturbances, as depicted in Fig. 5. There are several structures to synthesize a feedforward controller. However, a constant gain is the most used in process control plants, as it often yields satisfactory improvements^23^.

### Automatic control in the SAG mill circuit

The proposed automatic control system is designed to reduce the specific energy consumption of the SAG mill by minimizing the variability of a key process variable: the mill load. The system leverages an advanced regulatory control (ARC) structure, composed of a cascade control loop combined with a feedforward compensation, to address the complex, nonlinear, and multivariable nature of the process.

In the cascade configuration, the mill load serves as the controlled variable of the outer loop, which adjusts the setpoint of the inner loop—the fresh feed rate to the mill. The inner loop, in turn, regulates the feeder speed to ensure the actual feed rate tracks the setpoint. This hierarchical arrangement enhances control performance in two key ways. First, the inner loop acts rapidly to correct short-term deviations in the fresh feed rate, caused by changing ore characteristics such as bulk density, moisture content, or particle size distribution. These properties introduce nonlinear behavior in the feed measurement, where the same feeder speed may yield varying feed rates. By promptly compensating for these inconsistencies, the inner loop isolates the outer loop from fast disturbances and maintains feed stability.

The outer loop operates on a slower timescale, focusing on regulating the mill load—an inherently nonlinear function of feed rate, circulating load, and ore properties. It adjusts the fresh feed rate setpoint to keep the mill load near its optimal value, smoothing the impact of medium-term disturbances. This separation of control dynamics improves overall system stability and responsiveness.

To further enhance robustness, the system includes a feedforward controller that anticipates the effect of the circulating load, a measurable but highly variable disturbance resulting from crusher operation. Variations in the circulating rate directly affect the total mill feed and, consequently, the mill load. The feedforward element compensates for this disturbance by modifying the fresh feed rate setpoint in real time, reducing the corrective burden on the feedback loops and improving control accuracy.

**Fig. 6:**
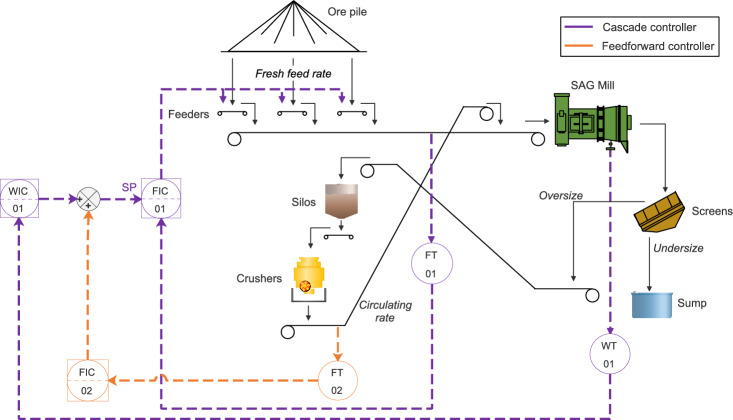
Proposed automatic control system (FT: Flow transmitter, WT: Weight transmitter. FIC: Flow indicator and controller, WIC: Weight indicator and controller).

Overall, this integrated ARC strategy leverages structural decomposition of control objectives—fast feed stabilization and slower load regulation—while incorporating measured disturbances into the control action. This design provides an effective and practical solution for handling the nonlinearities, time-scale separations, and interactions between variables that characterize SAG mill circuits. Figure 6 illustrates the proposed automatic control system applied to the SAG mill circuit.

## Industrial application

### Previous conditions

**Fig. 7:**
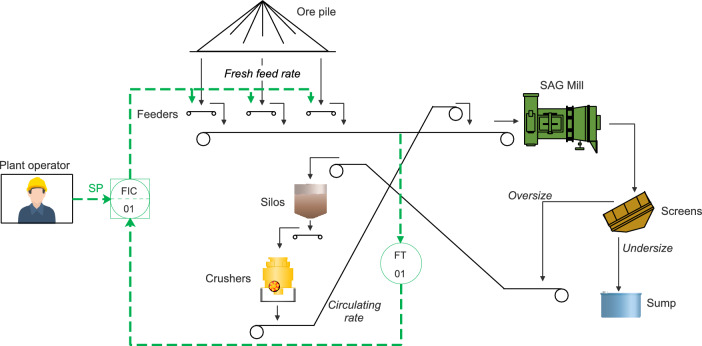
Manual control of the SAG mill circuit.

Figure 7 depicts how the control of the SAG mill circuit was conducted before the implementation of the proposed control strategy. The plant operator manually defined the fresh feed rate setpoint of a PID controller that regulates the speed of the feeders.

Despite the load setpoint value, the mill load was not considered for control purposes. Instead, it was only regarded by the operator as a warning flag: when the mill load considerably deviated from the setpoint, reaching value levels that could compromise production or the equipment, only then did the operator adjust the fresh feed rate setpoint to ensure the mill load returned to a proper operating level. This approach was production-driven, as the fresh feed rate is regarded as the production metric of this circuit. Hence, maximizing the fresh feed rate was prioritized over stabilizing the mill load.

### Control system implementation

The proposed control system was implemented in an ABB 800xA programmable logic controller (PLC), fully integrated into the plant’s distributed control system (DCS). The system architecture consists of a cascade controller with a feedforward compensator, designed to regulate the SAG mill load by manipulating the fresh feed rate.

In the cascade structure, the outer-loop controller (WIC-01) uses the mill load (WT-01) as the controlled variable ($y_1$), and its manipulated variable ($u_1$) is the fresh feed rate setpoint. The inner-loop controller (FIC-02) regulates the actual fresh feed rate (FT-02) by adjusting the speed of the feeders, which is the manipulated variable ($u_2$), making the fresh feed rate its controlled variable ($y_2$). This architecture allows the inner loop to quickly counteract disturbances from ore variability, ensuring that the fresh feed rate requested by the outer loop is precisely achieved in real time.

To synthesize the inner-loop controller, a first-order plus dead time (FOPDT) model was identified from historical plant data using an open-loop step test and the least-squares method^24^. The resulting model is:

2 $$\begin{aligned} G_2(s) = \frac{1.71}{30s + 1}e^{-58s}. \end{aligned}$$

Based on this model, a PI controller was initially tuned using the Internal Model Control (IMC) method^25^, with a filter factor of $\lambda = 58.7$, aiming for fast disturbance rejection in the inner loop.

A second FOPDT model was then identified to represent the dynamics between the fresh feed rate and the SAG mill load, again using a step test and the least-squares method:

3 $$\begin{aligned} G_1(s) = \frac{3.40}{100s + 1}e^{-47s}. \end{aligned}$$

From this model, a PI controller for the outer loop was designed using the IMC method with $\lambda = 98.0$. Due to the presence of measurement noise in the mill load signal, no derivative action was used, to avoid amplifying noise and producing aggressive control responses.

After the implementation of both loops in the PLC, a fine retuning procedure was carried out manually through limited trial-and-error adjustments. This step involved only minor refinements to the initially synthesized parameters, aiming to better align controller responsiveness and robustness with the specific operating conditions of the plant. These adjustments were minimal, as the initial IMC-based tuning already provided a solid baseline. Table 1 summarizes the final tuning parameters.

**Table 1:** Tuning parameters of the proposed controllers.

Controllers	Cascade		Feedforward
	Outer loop	Inner loop	
Parameters	$K_p$ = 0.20	$K_p$ = 0.15	$K_{ff}$ = 0.35
	$T_i$ = 100.00	$T_i$ = 30.00	

For the feedforward compensator, we adopted a simple yet effective approach using a gain-only formulation, in line with tuning guidelines such as those from^26^. After empirical testing, a gain factor of 0.35 was selected. This value was determined based on its ability to smooth the compensation effect on the fresh feed rate setpoint in response to changes in the circulating load. This strategy mitigates the tendency of coarse particles in the circulating load to increase the mill load, thus reducing unnecessary variability and improving the overall performance of the control system.

### Results and comparison

**Fig. 8:**
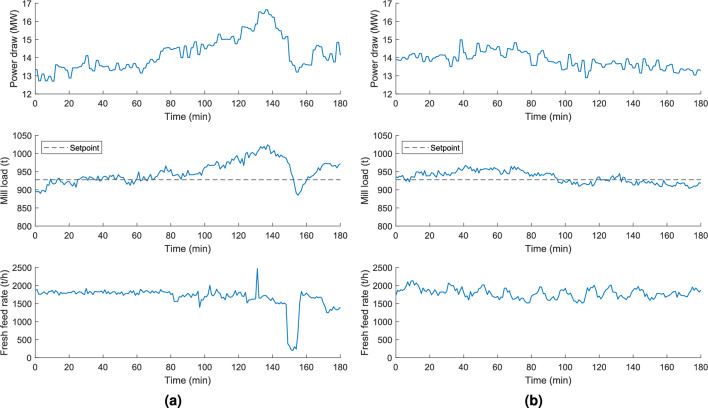
Variables behavior for comparison test between (**a**) manual and (**b**) automatic control.

In Fig. 8, a comparison test between manual and automatic control of the SAG mill showcases distinct behaviors in key process variables. Due to the nature of industrial operations, only one control strategy can be active at a time—either manual or automatic—making simultaneous testing infeasible. This limitation is common across most real-world applications of industrial control systems. To ensure a fair and meaningful comparison, both tests were conducted on the same day and under similar ore characteristics, as confirmed by on-site process engineers who closely monitored the test conditions. Importantly, no sensor faults or equipment failures were recorded during the test periods, reinforcing that the observed improvements are attributable to the implemented control strategy rather than to transient external factors.

During manual operation, the mill load fluctuated significantly. This variability can be attributed to the inherent limitations of human supervision, as operators are typically responsible for monitoring and responding to multiple process units simultaneously. As shown in previous studies, the cognitive workload, multitasking pressure, and lack of standardized interfaces in mineral processing control rooms limit operators’ ability to perform timely and consistent control actions^27,28^. Specifically, in the manual test, the operator maintained the fresh feed rate at approximately 1800 t/h under the assumption that this fixed value would ensure stable operation. While this worked reasonably well during the first 100 minutes, the mill load began to detach from the setpoint thereafter, remaining elevated until around minute 150—a 50-minute interval of inefficient operation. The operator then sharply reduced the feed rate to around 300 t/h to regain control. After minute 160, the operator attempted to return to the original value of 1800 t/h but quickly readjusted to 1500 t/h, recognizing the initial rate was inadequate. This sequence illustrates both the reactive nature of manual control and the difficulty of maintaining stable operation through fixed, human-driven setpoints.

In contrast, the proposed control strategy continuously regulates the fresh feed rate based on real-time feedback, resulting in a significant reduction in mill load and power draw variability. It is important to note that the fluctuations observed in the fresh feed rate under automatic control are not a sign of instability, but a necessary mechanism to maintain the mill load as close as possible to its setpoint. These fluctuations compensate not only for variations in the circulating load but also for inherent changes in ore characteristics. Therefore, even though the fresh feed rate may appear more variable than in manual mode, this variability reflects the system’s adaptability and responsiveness—attributes that enable the mill to operate in a more efficient regime for a greater proportion of time.

**Fig. 9:**
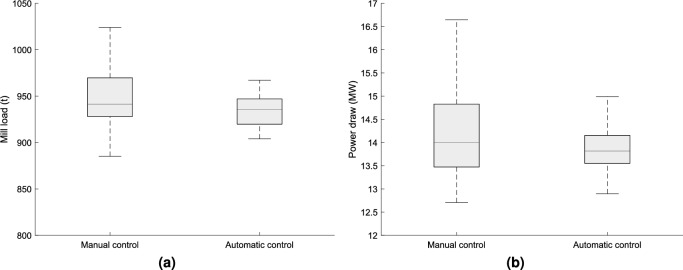
Variability comparison of the mill (**a**) load and (**b**) power draw.

As illustrated in Fig. 9, this test demonstrated a significant decrease in mill load and power draw variability through the implementation of the proposed automatic advanced regulatory control structure. The standard deviation of the mill load decreased from 30.92 t under manual control to 16.38 t under automatic control, representing a reduction of 47.02%. Consequently, the variability in power draw also decreased from 0.94MWh to 0.44MWh, a reduction of 53.19%.

The proposed automatic control system underwent a three-month testing period, during which the circuit’s average specific energy consumption was 8.75 kWh/t. For comparison, data from the three months preceding implementation—under manual operation—showed an average of 9.29 kWh/t. This sustained reduction of 5.84% in specific energy consumption, along with a 1.90% increase in productivity, reinforces the effectiveness of the control strategy. If the observed improvements had been primarily caused by temporary factors such as equipment behavior, sensor reliability, or operational anomalies, similar performance could not have been consistently maintained over this extended period. Therefore, the consistent results over time further support that the performance gains are attributable to the implemented automatic control system, rather than circumstantial differences between operational modes.

## Conclusion

This paper presented the design, implementation, and evaluation of an automatic control system for a semi-autogenous grinding (SAG) mill in an industrial copper plant. The control architecture, based on a combination of cascade and feedforward strategies, was developed to reduce variability in the mill load—a critical process variable directly influencing both energy consumption and grinding efficiency.

By maintaining the mill load close to its optimal value, the proposed system reduced the circuit’s specific energy consumption by 5.84% and increased productivity by 1.90%. These improvements are a direct consequence of operating the SAG mill in a region where breakage efficiency is maximized and unnecessary energy losses due to overfilling or underloading are minimized. Additionally, the system’s energy efficiency gains translate to a monthly avoidance of 94.72 tonnes of $CO_2$ emissions.

Beyond the technical results, this work highlights how engineers can leverage standard instrumentation and existing automation infrastructure to implement cost-effective and replicable solutions for sustainable process optimization. The proposed control strategy is simple to integrate into industrial control systems and shifts the operator’s role from manual regulation to strategic supervision. Ultimately, this study reinforces the notion that environmental responsibility and operational efficiency are not conflicting goals, but rather complementary outcomes of well-engineered control solutions.

As future work, two promising improvements are identified to enhance the proposed control strategy. First, the integration of an online particle size distribution (PSD) measurement into the feedforward control could allow dynamic adjustment of the fresh feed rate based on ore characteristics. Since coarser particles typically require more energy for grinding, incorporating PSD data would enable the system to reduce feed rate accordingly, preventing overload and improving energy efficiency. Second, the implementation of a reinforcement learning (RL) algorithm is proposed to determine the optimal mill load setpoint. Currently, this setpoint is fixed based on equipment specifications and operational heuristics. However, slight adjustments to the load—depending on the ore type—could lead to increased productivity. An RL-based approach could continuously learn from plant performance to identify and adapt the optimal operating point.

## Data Availability

The datasets generated and/or analysed during the current study are not publicly available due to industry regulations but are available from the corresponding author on reasonable request.
